# Influence of the Male Ejaculate on Post-Mating Prezygotic Barriers in Field Crickets

**DOI:** 10.1371/journal.pone.0046202

**Published:** 2012-10-10

**Authors:** Erica L. Larson, Jose A. Andrés, Richard G. Harrison

**Affiliations:** 1 Department of Ecology and Evolutionary Biology, Cornell University, Ithaca, New York, United States of America; 2 Department of Biology, University of Saskatchewan, Saskatoon, S.K., Canada; University of Melbourne, Australia

## Abstract

Post-copulatory interactions between males and females involve highly coordinated, complex traits that are often rapidly evolving and divergent between species. Failure to produce and deposit eggs may be a common post-mating prezygotic barrier, yet little is known about what prevents the induction of egg-laying between species. The field crickets, *Gryllus firmus* and *G. pennsylvanicus* are isolated by a one-way reproductive incompatibility; *G. pennsylvanicus* males fail to fertilize *G. firmus* eggs or to induce normal egg-laying in *G. firmus* females. We use experimental crosses to elucidate the role of accessory gland-derived vs. testis-derived components of the *G. firmus* male ejaculate on egg-laying in conspecific and heterospecific crosses. Using surgical castrations to create ‘spermless’ males that transfer only seminal fluid proteins (SFPs) we test whether *G. firmus* male SFPs can induce egg-laying in conspecific crosses and rescue egg-laying in crosses between *G. pennsylvanicus* males and *G. firmus* females. We find *G. firmus* SFPs induce only a small short-term egg-laying response and that SFPs alone cannot explain the normal induction of egg-laying. *Gryllus firmus* SFPs also do not rescue the heterospecific cross. Testis-derived components, such as sperm or prostaglandins, most likely stimulate egg-laying or act as transporters for SFPs to targets in the female reproductive tract. These results highlight the utility of experimental approaches for investigating the phenotypes that act as barriers between species and suggest that future work on the molecular basis of the one-way incompatibility between *G. firmus* and *G. pennsylvanicus* should focus on divergent testis-derived compounds or proteins in addition to SFPs.

## Introduction

Traits that mediate interactions between males and females are critical for reproduction and yet often evolve rapidly and are highly divergent between species. Therefore, these traits may be particularly important in the early divergence of isolated populations and in speciation [1]–[6]. Although we are often struck by the diversity of conspicuous behaviors involved in courtship and mate recognition, post-copulatory interactions between males and females are equally diverse and complex [7], [8]. As a result, fertilization in a heterospecific cross can fail at a number of critical steps, resulting in post-mating prezygotic barriers between species. These barriers can range from traits that prevent sperm and eggs from meeting (e.g. sperm transfer, sperm storage, sperm utilization, egg-laying, sperm binding) to intracellular traits that prevent the sperm nucleus and egg nucleus from fusing (e.g. incomplete sperm entry, sperm folding) [6], [9]–[11].

**Figure 1:**
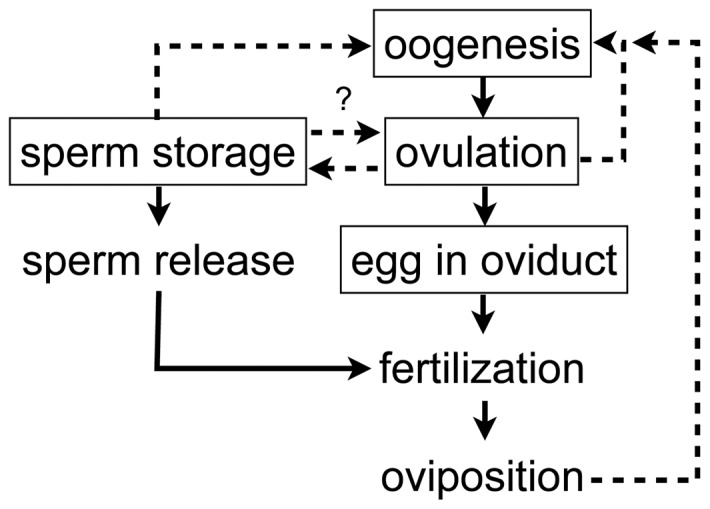
Egg-laying in female insects. Diagram showing the steps involved in egg-laying in female insects (solid lines) and the interactions that may stimulate increased egg-laying (dashed lines). In boxes are steps that may be mediated by seminal fluid proteins. The interaction between sperm storage and ovulation is hypothesized based on the observation that sperm transfer may increase ovulation and sperm depletion, thereby affecting the number of eggs laid. The figure is re-drawn from Bloch Qazi et al. (2003).

Egg-laying in insects provides an example of the complexity of male-female interactions. Egg-laying is a multi-step process that involves egg production within the ovary (oogenesis), release of the egg from ovary into the oviducts (ovulation), progression of the egg down the oviducts, union of the sperm and egg within the genital chamber (fertilization) and the deposition of the egg into a particular substrate (oviposition). These steps are tightly linked to the proper transfer and storage of the male ejaculate. Oogenesis is increased when sperm is stored within the female storage organ, and ovulation interacts with sperm storage and sperm release from storage to facilitate successful fertilization (Fig. 1, reviewed in [12]). A reduction in the efficiency or a failure at any of these steps can lead to reproductive incompatibilities in insects.

The failure of the male ejaculate to stimulate egg-laying between species has been observed in fruit flies [13], [14], beetles [15], katydids [16], lacewings [17] and ground crickets [18]. A similar phenomenon is sometimes observed between populations within a single species [19], [20]. Failure to produce and deposit eggs may be a common post-mating prezygotic barrier, yet little is known about what prevents the induction of egg-laying between species. We do know which components of the male ejaculate (testis-derived vs. accessory gland- derived) induce intraspecific egg-laying in a variety of insect species [21], and the molecular interactions of the male ejaculate and female reproductive tract that induce egg-laying in *Drosophila melanogaster* are now well understood [7], [8], [12], [22]. By characterizing the intraspecific mechanisms that result in egg-laying, we can begin to make inferences about how egg-laying breaks down between species. Here, we use experimental crosses both within and between species to test the influence of components of the male ejaculate on egg-laying and fertilization between two closely related species of field cricket.

### Post-mating prezygotic barriers in field crickets

The field crickets, *Gryllus firmus* and *G. pennsylvanicus*, are recently diverged species (<0.5% mtDNA divergence [23]) that interact in a hybrid zone in the northeastern United States, extending from Massachusetts south into Virginia [24]. The cricket species have diverged both ecologically [25]–[28] and behaviorally [29]–[31], but an important barrier between these species is a one-way incompatibility between *G. firmus* females and *G. pennsylvanicus* males [32]. Despite normal sperm transfer and storage, *G. firmus* females mated with *G. pennsylvanicus* males do not produce fertilized eggs [11], [33]. Fertilization appears to break down in this cross somewhere between the release of sperm from storage and the sperm entering the egg [11]. There is an equally striking reduction in egg-laying for these females. A *G. firmus* female mated with conspecifics will lay approximately 700 eggs over her lifetime, while a virgin female will produce less than 50 eggs and typically only late in life. *Gryllus firmus* females mated with *G. pennsylvanicus* males will lay about twice the number of eggs as virgin females, but significantly fewer eggs than a female mated to a conspecific [31]. In contrast, the reciprocal cross produces viable, fertile offspring in numbers indistinguishable from conspecific matings [31], [32].

Seminal fluid proteins (SFPs), which are synthesized and secreted from the male accessory gland and transferred to females during copulation, are known to play a role in many of the processes that may underlie a breakdown in egg-laying and fertilization, including ovulation, sperm storage, and sperm release (Fig. 1). Accessory gland genes from *G. firmus* and *G. pennsylvanicus* have been characterized through transcriptome sequencing [34], [35] and proteomics of the seminal fluid [36]. Many of the SFPs are found to be rapidly evolving under positive selection. However, there is currently no direct functional link between the divergence we observe in *Gryllus* SFPs and the post-mating prezygotic barriers that isolate these taxa. One step towards exploring this connection is to characterize the intraspecific and interspecific mechanism(s) that induce egg-laying and fertilization.

We attempt to elucidate the roles of accessory gland-derived vs. testis-derived components of the male ejaculate on these two barriers by asking whether *G. firmus* male SFPs induce egg-laying in *G. firmus* females and can “rescue” the cross between *G. firmus* females and *G. pennsylvanicus* males. We test the influence of *G. firmus* SFPs by mating females to surgically castrated (“spermless”) conspecific males that transfer only SFPs. We find that SFPs induce only a modest short-term egg-laying response and that SFPs alone cannot explain the normal induction of egg-laying. We also try to rescue the incompatibility by mating *G. firmus* females to males of both species. Again, we find no evidence that *G. firmus* SFPs can rescue the one-way incompatibility.

## Materials and Methods

### Cricket Collections

We collected crickets in August of 2006, 2009 and 2011 from pure populations of *G. firmus* in Guilford, CT, USA (N 41°16′9″; W 72°39′59″); near Hammonasset Beach State Park, CT, USA (N 41°16′4″; W 72°34′14″); and Milford, CT, USA (N 41°11′48″; W 73°4′30″) and *G. pennsylvanicus* in Ithaca, NY, USA (42°24′35″; −76°32′46″). Crickets were collected as late instar nymphs, separated by sex and maintained in large laboratory colonies with food (cat and rabbit food), water vials and egg flats for shelter, under a 12∶12 h light/dark cycle at 28°C. Every two days we isolated crickets that had become adults and maintained them in same-sex groups of 6–8 crickets in plastic containers (30×16×9 cm). No specific permits were required for the described collections because the study organisms are not endangered or protected species and the collection locations are not privately own or protected.

### Matings

For each experiment, virgin adult crickets between 6–10 days post-eclosion were randomly assigned to treatment (described below). We abbreviate treatments to indicate species (F = *G. firmus*, P = *G. pennsylvanicus*) and the order females were mated, with the first letter representing the female and subsequent letters representing the males with which she mated (*e.g.*, FFP represents a *G. firmus* female that mated with a *G. firmus* male followed by a *G. pennsylvanicus* male). Subscripts represent specific manipulations of male crickets described in the following section (*e.g.* FF_C_ represents a *G. firmus* female mated with a *G. firmus* male that was surgically castrated).

For each cross, cricket pairs were placed in petri dishes (9 cm) lined with moistened filter paper to provide traction. We considered matings complete when the male was observed to successfully transfer and properly attach the spermatophore to the female genital opening. To standardize the spermatophore attachment time and allow the spermatophore contents to be transferred completely, we left mated pairs undisturbed, allowing males to guard females and prevent early spermatophore removal. After males reinitiated courtship (approximately 45 min) we removed males from the mating chamber and females were either presented with a second male or were isolated in individual chambers depending on the experiment. Females that did not mate within 60 minutes of adding either a first or second male to the mating chamber were removed from the experiment.

Following matings, females were isolated in individual containers (30×16×9 cm) and provided with food, water, shelter and a petri dish (9 cm) filled with a mixture of moistened sand and soil as oviposition substrate. Food and water were replaced twice a week, oviposition substrate was periodically moistened, and mortality was scored every two days.

### Surgical Castrations

**Figure 2:**
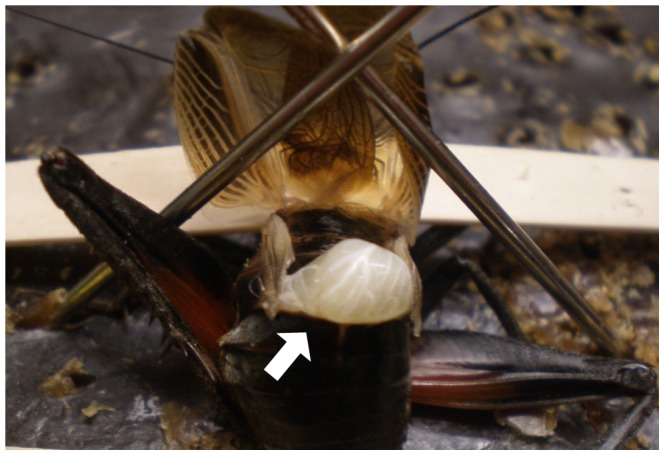
*Gryllus firmus* male undergoing a surgical castration. The incision is made between the dorsal 2^nd^ and 3^rd^ segments, and each testis (arrow) is gently removed.

To isolate the roles of testis-derived and accessory gland-derived components of the male ejaculate, we created spermless males using surgical castrations. Surgeries were performed on adult males 5–6 days post-eclosion, after the cuticle had hardened (surgeries performed too soon after eclosion result in high mortality rates). Males were divided into two categories: males that would be surgically castrated (F_C_) and males that would undergo a sham castration (F_S_) to serve as a control for effects of surgery on the male ejaculate production or content. Prior to surgery, males were anesthetized by chilling at 4°C for at least 30 minutes. Using fine forceps, we made an incision across the dorsal side through the intersegmental membrane between the 2nd and 3rd abdominal segments and gently teased open the wound. For F_C_ males we completely removed each testis and severed the vas deferens (Fig. 2), while for F_S_ males we probed the wound and body cavity to try to mimic testis removal. We then sealed the wound with Vetbond™Tissue Adhesive (3 M, St. Paul, MN, USA), which polymerizes after contact with tissue and body fluids, binding the wound edges together. Following surgery, males were placed in a sterile petri dish with moistened cotton for water, and allowed two days to recover. Males in both categories had a high survival rate following surgery (F_C_ N = 83, 86.7% survived; F_S_ = 69, 89.8% survived). After the recovery period males resumed normal mate calling and courtship behaviors. Males were then transferred to individual containers and provided with food, shelter and water and the containers were cleaned every day.

Following the recovery period, males that were surgically castrated were placed in a petri dish with a single virgin *G. firmus* female, and allowed to mate repeatedly in order to deplete stores of mature sperm from the seminal vesicles. After two days of repeated matings, we checked males for remaining stored sperm by removing his spermatophore immediately after attachment to the female and examining the spermatophore contents under a compound light microscope (400×). Spermatophores were gently removed so that all components of the spermatophore remained intact, placed on a microscope slide in a drop of phosphate buffered saline (PBS), and then gently squashed with a coverslip. When normal spermatophores were observed in this manner, a viscous liquid could be observed evacuating the spermatophore tube, followed immediately by long, thread-like sperm. If sperm were observed, males were allowed to mate repeatedly for another eight hours and were checked again the following day. If males were depleted of stored sperm, the viscous seminal fluid was still observed evacuating the spermatophore tube. If no sperm were observed in a male's spermatophore for three consecutive days, we considered the male spermless. Males were kept a minimum of 4 days during which they mated only once a day; this treatment allowed full recovery from repeated matings. Of the 72 males that survived surgical castrations, 57 were successfully cleared of sperm.

To validate that surgically castrated males still transferred SFPs we used two-dimensional electrophoresis (2D–E) to visualize the protein content of the spermless ejaculates. We collected two independent samples of both spermless spermatophores and normal spermatophores in liquid nitrogen, for a total of 4 samples (N = 25 males per sample). We homogenized each sample in 100 ul of ice-cold PBS and centrifuged (14,000 rpm for 1 min at 4°C) to separate the ejaculate from most sperm and spermatophore debris. The 2D–E analysis, including sample preparation and quantification, first and second dimension separations using isoelectric focusing and Tris-SDS-PAGE electrophoresis, gel staining, image capture and analysis were carried out by the Cornell Core Laboratories Center for Proteomics and Mass Spectrometry.

### Influence of the G. firmus ejaculate on egg-laying and longevity

To test the effects of seminal fluids on egg-laying in *G. firmus* females, we measured the total number of eggs produced by *G. firmus* females that 1) remained unmated (F = 57), 2) were mated to *G. firmus* males that were surgically castrated (FF_C_ = 56), 3) *G. firmus* males that underwent a sham castration (FF_S_ = 61), and 4) normal *G. firmus* males (FF = 60). By surgically castrating the *G. firmus* males, we could compare the effects on egg-laying of SFPs alone with the effects of the complete male ejaculate.

In other insects (including crickets) components of the male ejaculate typically elicit a short-term egg-laying response within 24 h of mating. To estimate both the initial egg-laying response and a female's lifetime fecundity, we collected oviposition substrates at both 48 h following mating and at the end of the female's lifespan. We collected substrates at 48 h to allow each female time to adjust following transfer to a new container and provide sufficient time for egg-laying; *G. firmus* females that are frequently disturbed are less inclined to oviposit (EL Larson personal observation). Eggs were separated from the oviposition substrate using a series of sieves and we counted the total number of eggs for each time point (within 48 h and after 48 h).

### Influence of the G. firmus ejaculate on the one-way incompatibility

We performed two experiments to test whether the presence of a *G. firmus* male's ejaculate within the female reproductive tract could “rescue” the incompatibility (reduced egg-laying, no fertilization) between *G. firmus* females and *G. pennsylvanicus* males. For the first experiment *G. firmus* females were either mated to a normal *G. firmus* male immediately followed by a *G. pennsylvanicus* male (FFP = 9) or were mated first to a *G. pennsylvanicus* male followed by a normal *G. firmus* male (FPF = 11). Females were provided with oviposition substrate immediately after mating, and then allowed to oviposit for three weeks. Eggs were incubated at 28°C for 21 days and then at 4°C for 102 days to break diapause conditions and ensure synchronous hatching [32]. Eggs were then removed from chilled conditions and incubated at 28°C until hatching (approximately 17 days). We collected all hatchlings (1^st^ instar nymphs) each morning until all eggs hatched and stored nymphs at −80°C for paternity analysis. We randomly selected 20 nymphs per cross for genotyping.

For paternity analysis, we used highly polymorphic microsatellite markers (PGI, Gr143, G3 and G28). Two of these loci were developed from *G. pennsylvanicus*, and have been previously described [11]. The remaining two loci were developed from *G. firmus* using methods described in Hamilton et al. [37] and Larson et al. [11] with the addition of an enrichment by hybridization with biotinylated dimeric, trimeric and tetrameric nucleotide repeats. We quantified genetic variation for these new loci in one population of each species (*G. pennsylvanicus*: Ithaca, NY, USA; *G. firmus*: Guilford, CT, USA) (Table 1). Tests for linkage disequilibrium and deviation from Hardy-Weinberg equilibrium were performed using Genepop v. 4.1 [38] and we adjusted significance thresholds using the false discovery rate procedure [39]. Cervus v 3.0 [40] was used to test parentage exclusion probabilities, estimate null alleles and the polymorphic information content of the markers.

**Table 1:** Primer sequences and amplification conditions for *Gryllus* microsatellite loci used in paternity analysis.

Locus	Primer sequence (5′-3′)	T_a_	Size	Sp	N	N_a_	H_O_	H_E_	PIC	Null	GenBank
PGI	GAATGCATACATCAGTGTCATGAACA	56	220–	*Gf*	27	21	0.741	0.930	0.907	0.101	JN379460
(ATT_15_)	TGACTCAAAATAAGCATTATTTCAGC		334	*Gp*	14	15	0.929	0.939	0.898	0.011	
Gr143	CTGCCGCATTCACCAATCATTCAACTAT	58	150–	*Gf*	27	13	0.852	0.898	0.870	0.019	JN375328
(TG_11_)	CAACCAAGGGGCAAAATGAGTCAAACTT		204	*Gp*	14	9	0.857	0.820	0.763	0.038	
Gr3	GCGCGGCGACCGACTATTG	65–	153–	*Gf*	27	17	0.889	0.933	0.909	0.015	JX050157
(TG_16_)	CTCGCACCCTGTTAACAGTACTATCAAAAC	55	208	*Gp*	14	14	1.000	0.931	0.889	0.056	
Gr28	GCACCGCCCTAAACCCACGAC	65–	360–	*Gf*	27	6	0.667	0.648	0.762	0.046	JX050156
(TG_11_)	GGCACGGCAGCTTAAGGACATCAA	55	399	*Gp*	14	8	0.500	0.728	0.657	0.190	

T_a_ = annealing temperature (°C); Size = allele size range in base pairs; Sp = species; N = number of individuals scored; N_a_ = number of alleles; H_O_ = observed heterozygosity; H_E_ = expected heterozygosity; PIC = polymorphic information content; Null = frequency of null alleles.

Parental genomic DNA extractions from single femurs were performed using the DNeasy Blood and Tissue Kit (QIAGEN Inc., Valencia, CA, USA); offspring genomic DNA was extracted from entire nymphs using the DNAdvance Genomic DNA Isolation Kit (Agencourt, Beverly, MA, USA). The forward primer of each primer pair was labeled with a 5′ fluorescent tag (6-FAM, PET, NED, or VIC). We amplified these microsatellite loci using the Type-it Micosatellite PCR Kit (QIAGEN) following manufacturer's protocol with the addition of a touchdown protocol of 28 cycles of 95°C for 30 s, 59–53°C for 90 s (the annealing temperature decreased by 1°C each cycle for the first 6 cycles and remained at 53°C for the remaining 22 cycles) and 72°C for 30 s. Fluorescent PCR products were diluted 1∶15 in water, mixed with formamide and Genescan LIZ-500 size standard (Applied Biosystems Inc. Foster City, CA, USA) and run on an ABI Automated 3730 DNA Analyzer at the Cornell University Life Sciences Core Laboratories Center (CLC). Alleles were called using Genemapper (Applied Biosystems) and then verified by eye. The high level of polymorphism in our markers allowed us to assign paternity by eye.

For the second experiment *G. firmus* females were either mated to a surgically castrated male *G. firmus* male immediately followed by a *G. pennsylvanicus* male (FF_C_P = 3), a *G. pennsylvanicus* male immediately followed by a surgically castrated *G. firmus* male (FPF_C_ = 12), or a normal *G. pennsylvanicus* male (FP = 10). After mating, females were provided with oviposition substrate and allowed to oviposit for 48 h. We then counted the total number of eggs laid by each female.

### Statistics

To investigate the influence of *G. firmus* SFPs on *G. firmus* female egg-laying within 48 h of mating and after 48 h of mating, we constructed generalized linear mixed model (GLMM) contrasts to test the following hypotheses: Model 1: females mated with normal males (FF) will lay more eggs than females mated with males that that underwent sham castration (FF_S_); Model 2: females mated with surgically castrated males (FF_C_) will lay more eggs than unmated females (F); and Model 3: females in treatments without sperm (F, FF_C_) will lay fewer eggs than females in treatments with sperm (FF, FF_S_). Differences between collecting locations were controlled by including a random effect of population identity. Using the R package ‘lme4’ [41], we modeled the proportion of females that laid eggs within 48 h with GLMMs fitted with a binomial error structure and a logit link function and the male treatment as the predictor. The egg-count data were highly over-dispersed; therefore, we modeled the total number of eggs laid by *G. firmus* females after 48 h using GLMMs with a Poisson distribution, individual-level random effects [42], and male treatment as the predictor. To compare longevity of females between mating treatments we estimated survival curves using the Kaplan-Meier method and compared differences between treatments using the log-rank test in the R package ‘survival’ [42].

To test the influence of the *G. firmus* ejaculate on the one-way incompatibility, we modeled the effect of male species on the proportion of offspring sired by the second male (P_2_) for *G. firmus* females mated sequentially to both *G. firmus* and *G. pennsylvanicus* males. We constructed a GLM with binomial error structure and a logit link function using P_2_ as the response variable and the male species as the predictor. For the second experiment involving *G. firmus* females mated with surgically castrated *G. firmus* males and *G. pennsylvanicus* males no statistics were required to interpret the results. Figures were constructed using the R packages ‘plotrix’ [43] and ‘gplots’ [44]. All analyses were performed using the statistical package R version 2.12.0 [45].

## Results

### Seminal fluid protein content of spermless spermatophores

**Figure 3:**
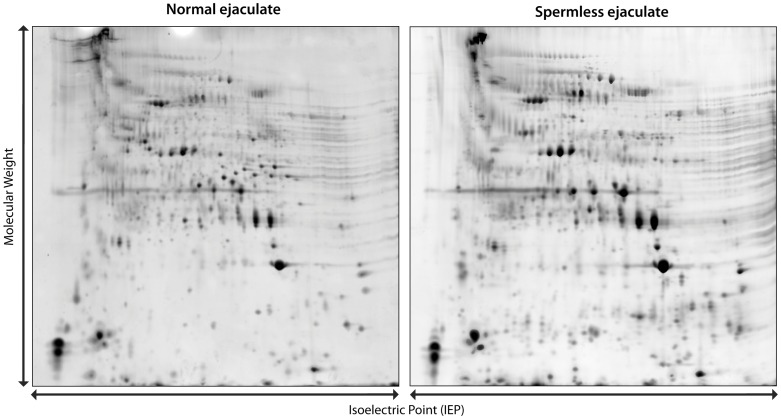
Two-dimensional gel electrophoresis of male ejaculates. Protein gels of a normal male ejaculate (left) and a spermless male ejaculate (right). Spermatophore samples were ground and centrifuged to remove spermatophore debris and sperm. Proteins were separated based on their isoelectric point in the first dimension and molecular weight in the second.

Representative examples of the 2D–E gels for the normal ejaculates (with sperm removed via centrifugation) and a spermless ejaculates are presented in Figure 3. Each spot represents a protein isoform. Overall, we estimate that there are about 630 protein spots present in all four samples. The patterns seen for normal and spermless spermatophore are very similar. Extra spots seen in the normal spermatophore extracts are presumably due to contamination from residual sperm.

### Influence of the G. firmus ejaculate on egg-laying

**Figure 4:**
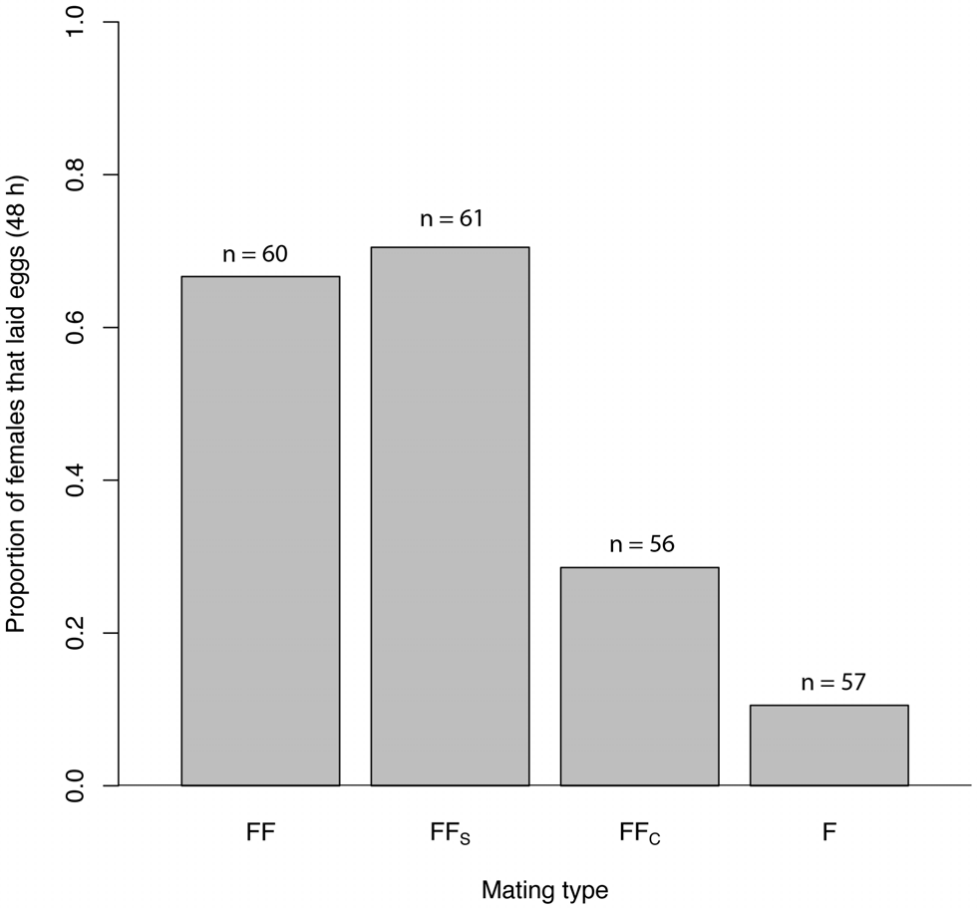
Proportion of *G. firmus* females that laid eggs within 48 h of mating. Females were mated with (1) a normal *G. firmus* male (FF), (2) a *G. firmus* male that underwent sham testes removal surgery (FF_S_), (3) a *G. firmus* male surgically castrated (FF_C_) or (4) remained unmated (F).

**Figure 5:**
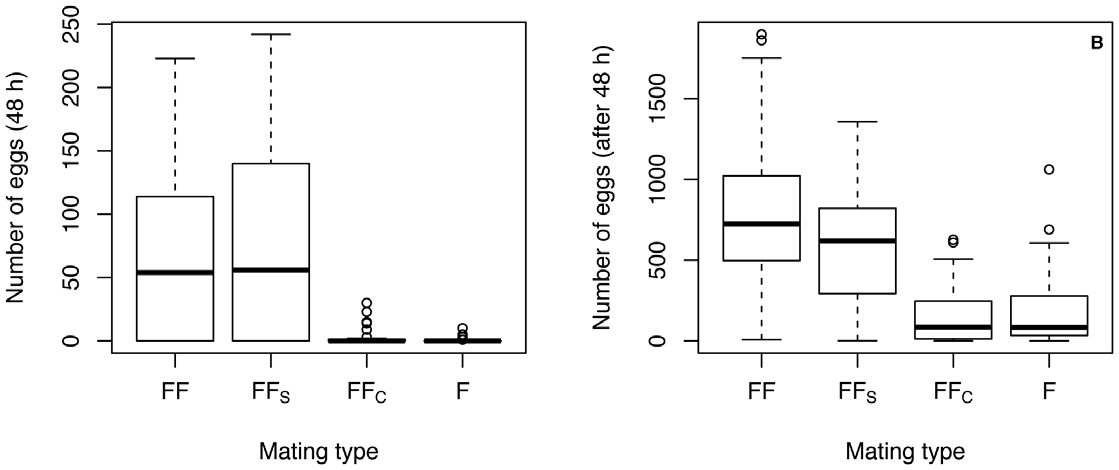
*Gryllus firmus* female egg production. Box plot of egg production **A**) 48 h following mating and **B**) total lifetime for *G. firmus* females that (1) mated with a normal *G. firmus* male (FF), (2) mated with a *G. firmus* male that underwent sham testes removal surgery (FF_S_), (3) mated with a *G. firmus* male surgically castrated (FF_C_) or (4) remained unmated (F).

When sperm are transferred to females (FF vs FF_S_), there is no effect of sham castration on either the proportion of females that laid eggs within 48 h (Fig. 4, GLMM: z = −0.451, df = 6, p = 0.652) or the number of eggs laid after 48 h (Fig. 5, GLMM: z = −1.12, df = 6, p = 0.263). A greater proportion of females mated with surgically castrated males (FF_C_) laid eggs in 48 h compared to females that remained unmated (F) (GLMM: z = 2.442, df = 6, p = 0.015); however, there was no significant difference in the number of eggs laid after 48 h (GLMM: z = −1.38, df = 6, p = 0.167). Comparisons between females mated with males that transferred sperm (FF, FF_S_) and females that did not receive sperm (FF_C_, F) revealed that in the former group there was both a greater proportion of females that laid eggs within 48 h (GLMM: z = 7.155, df = 6, p = <0.001) and females laid more eggs after 48 h (GLMM: z = −10.15, df = 6, p = <0.001).

### Influence of the G. firmus ejaculate on female longevity

**Figure 6:**
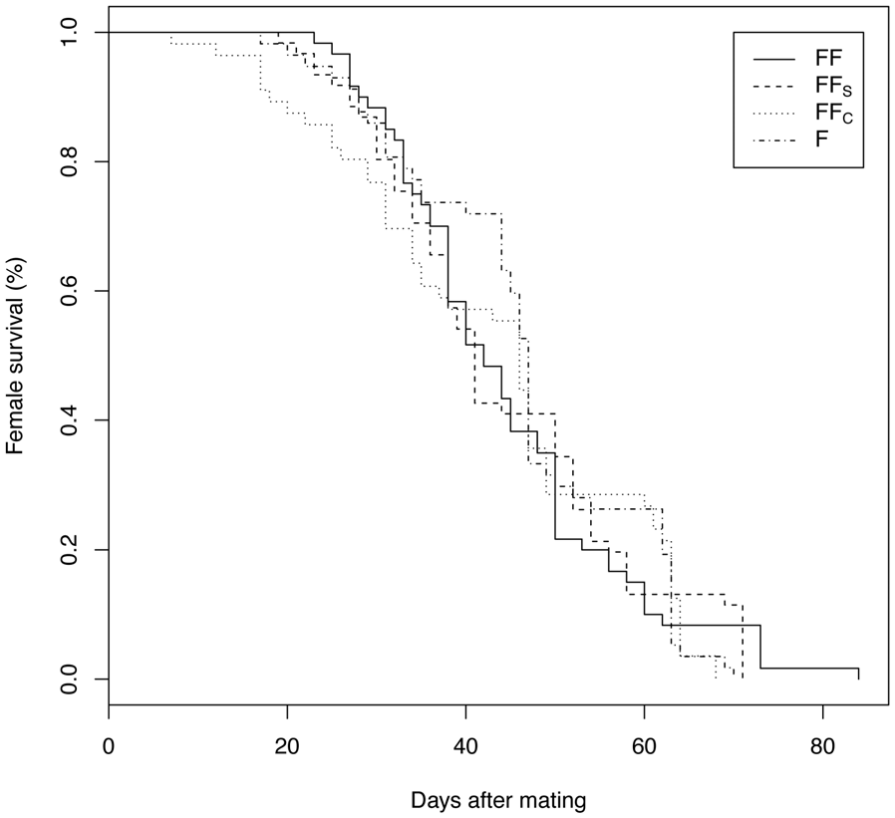
*Gryllus firmus* female longevity. Survivorship curves of *G. firmus* females (1) mated with a normal *G. firmus* male (FF), (2) mated with a *G. firmus* male that underwent sham testes removal surgery (FF_S_), (3) mated with a *G. firmus* male surgically castrated (FF_C_) or (4) remained unmated (F).

Female life span ranged from 7–84 days following mating (FF: 7–71; FF_S_: 17–84; FF_C_: 17–69; F: 17–73) with an average lifespan of 44 days (FF: 45.8; FF_S_: 41.8; FF_C_: 42.7; 47.6). There was no difference in lifespan among the four mating treatments (Fig. 6, log-rank: χ^2^ = 0.2, df = 3, p = 0.972).

### Influence of the G. firmus ejaculate on one-way incompatibility

None of the microsatellite loci used in this study deviated from Hardy-Weinberg equilibrium following false discovery rate correction and there was no evidence of linkage disequilibrium between any pair of loci (Table 1). Despite the presence of null alleles in two of these loci (PGI and G28), the combined nonexclusion probability of the second parent across all four loci was 0.004 and 0.006 for *G. firmus* and *G. pennsylvanicus*, respectively, indicating that these markers are appropriate for assigning paternity. Of the 474 nymphs selected for genotyping, 98.9% were genotyped and assigned paternity successfully. The remaining 1.1% had poor microsatellite amplification, most likely due to low quantities of DNA as a result of little starting material. We did not observe any non-parental alleles in the offspring. *Gryllus firmus* females mated sequentially to *G. firmus* and *G. pennsylvanicus* males (FFP, FPF) produced offspring that were sired only by *G. firmus* males regardless of mating order (t = −8.05×10^15^, df = 1, p = <0.001). *Gryllus firmus* females mated sequentially to surgically castrated *G. firmus* males and *G. pennsylvanicus* males (FF_C_P, FPF_C_) and *G. firmus* females mated only to *G. pennsylvanicus* males (FP) laid no eggs within a 48 h period.

## Discussion

### *Gryllus firmus* seminal fluid proteins induce only a marginal egg-laying response

Following mating, female insects undergo numerous physiological and behavioral changes. Pre-mating courtship or the mechanical stimulus of copulation can cause these changes, but the majority of changes are induced by components of the male ejaculate. In insects, sperm clearly play a critical role in fertilization, but many aspects of sperm function (viability, storage, activation, competition) and female response to mating (mating refractoriness, oogenesis, sperm utilization, ovulation, oviposition) are mediated by SFPs secreted from the male accessory glands or ejaculatory duct [7], [8], [21], [46], [47]. Seminal fluid proteins were first linked to these post-mating changes via whole tissue transplantation experiments in *Drosophila melanogaster*, where portions of the male accessory glands or testes were transplanted into the abdomens of virgin females [48], [49]. Subsequently, methods to isolate the roles of SFPs in fertilization have ranged from simple injections of SFP extracts into virgin females to the creation of “spermless” or “accessory glandless” males and the targeted knockdown of specific SFPs using RNAi [7], [8], [47].

Much of this effort has focused on how components of the male ejaculate elevate egg-laying in mated females. In *Drosophila*, where egg-laying is best understood, the male ejaculate alters a female's reproductive physiology over different timescales. Initially, there is a short-term increase in the number of eggs laid within the first 24 h of mating [50], [51]. This short-term response is induced by the presence of at least three SFPs in the female reproductive tract, sex peptide (SP, Acp70A), the prohormone ovulin (Acp26Aa) and CG33943 [51]–[55]. However, this response is transient, and the presence of both sperm and SFPs in the sperm storage organ is required to maintain elevated levels of egg-laying [56], [57]. This so-called sperm effect or long-term post-mating response was thought to be induced by sperm-binding receptors or stretch receptors within the female sperm storage organ [56]. It is now clear that the sperm effect, at least in *Drosophila*, is actually an SFP effect, mediated in part by the SFP sex peptide [53], [54]. Sex peptide binds to the tails of sperm and is slowly released from sperm within the female storage organ [22], [58], [59]. It appears that sperm may act as both carriers and reservoirs for SFPs, enabling sperm to reach target cells within the female reproductive tract and maintaining their effects on female reproduction over an extended period. At least four other SFPs have been identified that act in concert with sex peptide to sustain the long-term post-mating response, and at least one of these proteins also binds to sperm [55], [60].

*Drosophila* has been a model for understanding post-mating male and female interactions, and from this work it has become clear that seminal fluid proteins stimulate egg-laying in mated females but that to do so they must interact with sperm. However, this picture of reproduction appears to vary greatly across taxa. In other Dipterans there is evidence that SFPs alone can induce egg-laying in mated females [61], [62], whereas in Lepidoptera egg-laying is often triggered by the presence of eupyrene sperm in the spermatheca [63]–[65], but there is at least one case of SFPs inducing partial egg-laying [66]. There are few examples from the Coleoptera, but in at least two species both components of the testis/seminal vesicle and the accessory gland induce egg-laying, although the accessory gland extracts had a minimal influence [67].

In Orthoptera, the picture is even less clear. Egg-laying is stimulated by SFPs in some grasshoppers [68]–[72] and in ground crickets [73], while in at least one grasshopper the combination of mechanical stimulus and testis derived components can induce egg-laying [74]. In the field crickets, *Acheta domesticus* and *Teleogryllus commodus* egg-laying is initially induced by prostaglandins, autocrine hormones transferred to females as part of the seminal fluid [75]–[79], but the presence of sperm in the spermatheca is required to maintain long term egg-laying (similar to *Drosophila*) [80], [81]. Prostaglandins or prostaglandin precursors have been found to be synthesized in both the testes (*T. commodus* and *A. domesticus*) and the accessory glands (*A. domesticus* and *Locusta migratoria*) of Orthoptera, although the prostaglandins found in *L. migratoria* do not appear to be involved in egg-laying [82]. The only study to attempt to induce egg-laying in field crickets using whole ejaculatory-fluid extracts failed to see a response [83]. However, in that study ejaculate extracts were injected into the abdominal cavity and may have failed to elicit an egg-laying response because SFPs did not reach target receptors within the female reproductive tract.

Our use of castrated males to transfer SFPs to virgin females is a more effective way of delivering SFPs directly into the female reproductive tract while controlling for any effects of mating. Still, we found that SFPs without the presence of sperm or other testis-derived compounds induced only a modest short-term egg-laying response in the field cricket *G. firmus*. This response is small compared to egg-laying in normally mated females, a result in stark contrast to the induction of egg-laying seen in other taxa. In the long-term, there was no difference in the fecundity of virgin females and those that received SFPs. This suggests that SFPs indeed play some role in eliciting egg-laying behavior over the short term, but that testis derived factors are required for both the short and long term post-mating egg-laying response in *G. firmus*.

It might be argued that the “effectors” of egg-laying did not reach their targets in the female reproductive tract. However, we know that spermatophores of surgically castrated males transfer seminal fluid. Seminal fluid can be directly observed evacuating the spermatophore tube of spermless spermatophores (see Methods). Furthermore, analysis of this fluid using 2-D gel electrophoresis clearly reveals the same pattern of protein spots that are seen in extracts from normal spermatophores (Fig. 3).

There is also no reason to believe that the multiple matings required to create the spermless males adversely affect SFP volume or content. Field crickets are highly promiscuous and males mate repeatedly both in the wild and the laboratory [84]–[92]. Males will only re-mate when a fully formed spermatophore is present in their spermatophore pouch [93], [94]. As a result, both the timing and the frequency of matings in field crickets are dependent on spermatophore production, which in *Gryllus firmus* males is approximately every 45 minutes throughout the day [31], [84]. Both the spermatophore and the seminal fluid are composed of proteins secreted by the male accessory gland [95], [96] and it is unlikely that a male would have sufficient accessory gland function to produce a spermatophore, but not the seminal fluid proteins. There is also evidence that male insect ejaculate content is consistent across repeated matings [97] and throughout their lifetime [98], [99]. In our protocol, castrated males were mated repeatedly only during the first two days following surgery recovery. Subsequently, they only mated once a day for a minimum of four days, well below the expected number of matings for a male field cricket. Thus, our treatment should not compromise SFP production.

Given our observation of SFPs in the seminal fluid of spermless spermatophores and our delivery method of SFPs directly into the female reproductive tract (as opposed to abdominal injections), our failure to find any large or long term egg-laying response induced by SFPs suggests that SFPs alone are not sufficient to stimulate egg-laying in *G. firmus*. This is consistent with a similar failure of SFPs to induce egg-laying in *G. bimaculatus* [83]. It is possible, even very likely, that we see a failure of SFPs to induce egg-laying in field crickets because key SFPs bind to the sperm for transport into the female reproductive tract as has been demonstrated in *Drosophila* [22], [53], [54], [58], [60], [100].

The question of what components of the male ejaculate stimulate egg-laying is an important one, not simply for a better understanding of insect reproduction, but because these components, if diverged, may constitute a barrier to gene exchange between closely related taxa. To our knowledge, only one study, in *Drosophila pulchrella* and *D. suzukii*, has attempted to differentiate between the components of the male ejaculate that induce egg-laying in heterospecific crosses. In that case, a one-way incompatibility between *D. pulchrella* females and *D. suzukii* males is a result of both low sperm storage and severely reduced egg-laying. When *D. pulchrella* females are implanted with accessory gland tissue from conspecifics they have an ovulation rate that is 75% of a normally mated female, whereas females implanted with heterospecific accessory glands have an ovulation rate of only 54% [13], [101].

### Gryllus firmus seminal fluids do not affect female lifespan

Mating is often costly to females and results in decreased lifespan due to SFPs that are toxic to females. For example in *Drosophila*, females that receive SFPs during mating have a reduced lifespan [102], but these SFPs serve to increase male mating success [103]. In species, such as crickets, that are promiscuous and mate more often than is required for fertilization of their eggs, the male ejaculate may actually increase female lifespan [92], or have no effect [104]. In one case, female lifespan in the cricket *G. bimaculatus* was reduced as a result of the injection of SFPs into the female abdomen, but it is difficult to determine whether this is a normal effect of SFPs or a result of SFPs present in the body cavity where they may be toxic [83]. We found no effect of SFPs on female lifespan. This is consistent with similar studies in *Gryllus firmus* and *G. pennsylvanicus* that found no difference in the lifespan of singly mated, doubly mated and virgin females [31], despite the fact that virgin females and females mated with surgically castrated males are often ‘bursting’ with eggs (EL Larson personal observation). It is possible that there are lifespan benefits or costs to mating, but that a greater number of matings is required to see an effect in *G. firmus*.

### Gryllus firmus *seminal fluid proteins alone do not rescue the one-way incompatibility*

In *Drosophila*, sperm function is dependent on the presence of SFPs, and males that transfer only sperm (*prd* males) are completely sterile. However, females mated to *prd* males and subsequently mated to males transferring only SFPs (*tud* males) can occasionally lay fertile eggs [105]. The number of rescued crosses is very low (less than 1%). Nonetheless, this suggests that SFPs from one male can facilitate fertilization by the sperm of a second male. Alternatively, SFPs could act as a specific stimulus, only affecting sperm from the same male, or complementation may only be possible within species and SFPs may not interact with heterospecific sperm.

Our results suggest that in *G. firmus*, the latter is the case. The *G. firmus* male ejaculate was unable to facilitate fertilization for heterospecific sperm and *G. firmus* males sired all offspring in females mated sequentially with both species. Similar results have been observed in double matings of the lacewing species *Chrysopa quadripunctata* and *C. slossonae*, but in these taxa, there are fewer heterospecific sperm stored [17]. *Gryllus firmus* male SFPs also failed to induce egg-laying when *G. pennsylvanicus* sperm were present in the spermatheca. Therefore, failure of *G. firmus* SFPs alone to stimulate normal egg-laying in *G. firmus* females is not simply a result of mechanical stimulus (e.g., stretch receptors in the spermatheca). SFPs may need to act in concert with sperm to induce egg-laying in *G. firmus* females. Unfortunately, we are not able to test whether testis-derived compounds alone can induce oviposition. Surgical removal of accessory glands would prevent the formation of the spermatophore necessary to transfer sperm to a female.

### Conclusions

Our results highlight the utility of experimental approaches for investigating the phenotypes that act as barriers between species and provide new directions for investigating the molecular changes that lead to these barriers. The nature of the one-way incompatibility between *G. firmus* females and *G. pennsylvanicus* males suggests a role for SFPs, and both egg-laying and fertilization are traits that are often mediated by SFPs in other taxa. In addition, many SFPs are highly divergent between *G. firmus* and *G. pennsylvanicus* and appear to be evolving as a result of positive selection [34], [36]. Although the results of this study do not exclude a role for SFPs in these barriers, they do suggest that SFPs are not solely responsible for successful egg-laying. In particular, testis derived components, such as sperm or prostaglandins, either stimulate egg-laying or act as transporters for SFPs to targets in the female reproductive tract. Future work on the molecular basis of the one-way incompatibility between *G. firmus* and *G. pennsylvanicus* should focus on divergent testis-derived compounds or proteins.

A great deal of research on post-mating prezygotic barriers in internal fertilizers has concentrated on the role of SFPs [106]–[108], but our results suggest that focusing on SFPs alone is too narrow. Although there are now numerous examples of rapid divergence in SFPs between closely related species across diverse taxonomic groups [108] and there is some evidence of post-mating prezygotic barriers between several of these species [36], [109], there are few studies that provide a functional link between the rapid evolution of SFPs and post-mating prezygotic barriers. While documenting patterns of divergence between species is an important step, functional studies through experimental crosses are needed to determine whether divergent genes play a role in reproductive barriers between species.
